# Modeling the role of asymptomatics in infection spread with application to SARS-CoV-2

**DOI:** 10.1371/journal.pone.0236976

**Published:** 2020-08-10

**Authors:** Hana M. Dobrovolny

**Affiliations:** Department of Physics & Astronomy, Texas Christian University, Fort Worth, TX, United States of America; University of Surrey, School of Veterinary Medicine, UNITED KINGDOM

## Abstract

SARS-CoV-2 started causing infections in humans in late 2019 and has spread rapidly around the world. While the number of symptomatically infected and severely ill people is high and has overwhelmed the medical systems of many countries, there is mounting evidence that some of the rapid spread of this virus has been driven by asymptomatic infections. In this study, we use a compartmental mathematical model of a viral epidemic that includes asymptomatic infection to examine the role of asymptomatic individuals in the spread of the infection. We apply the model to epidemics in California, Florida, New York, and Texas, finding that asymptomatic infections far outnumber reported symptomatic infections at the peak of the epidemic in all four states. The model suggests that relaxing of social distancing measures too quickly could lead to a rapid rise in the number of cases, driven in part by asymptomatic infections.

## Introduction

In late 2019, a novel coronavirus (SARS-CoV-2) began transmitting in humans in Wuhan, China [1, 2] and has since spread widely around the world. The virus can cause a severe respiratory illness, known as COVID-19, characterized by fever and cough that can lead to respiratory failure and death [3, 4]. The virus appears to spread easily from human to human [5, 6], surviving well in aerosolized form and lasting for long periods of time on surfaces [7]. It has also become increasingly apparent that asymptomatic or unreported cases are playing a role in the rapid spread of the virus [8, 9].

A number of studies have investigated the possibility of asymptomatic carriers of SARS-CoV-2 and have tried to estimate their number using isolated clusters of cases. One study estimated that the asymptomatic proportion of SARS-CoV-2 infections on the Diamond Princess cruise ship docked outside Tokyo while an outbreak spread onboard was ∼18% [10]. A study of Japanese evacuees from Wuhan, China estimated the proportion of asymptomatic infections at ∼30% [11]. A similar study of German nationals evacuated from Wuhan found 2 of the 114 evacuees were asymptomatically infected with SARS-CoV-2 [12]. Testing of every person in an isolated Italian village revealed that 50-75% of people were asymptomatic [13]. Even in high-risk populations, there appear to be some asymptomatic cases. A study of a nursing home in King County, Washington found ∼30% of patients were asymptomatic on the day of testing for SARS-CoV-2, with ∼4% remaining asymptomatic upon followup a week later [14]. A study in hospitalized patients in Beijing found that ∼5% of patients testing positive for SARS-CoV-2 had asymptomatic infections [15]. While the exact proportion of asymptomatic infections is still unclear, these studies indicate that asymptomatic infections with SARS-CoV-2 are not uncommon.

However, it is not enough that asymptomatic infections exist. In order for asymptomatic individuals to spread the infection, they must be able to shed and transmit the virus to others. There are some studies that indicate this might in fact be the case for asymptomatic infections with SARS-CoV-2. Zhang et al. [16] report on a familial cluster of COVID patients initiated by an asymptomatic individual. There is also a second case report of a different asymptomatic individual infecting other members of his family [17]. A series of SARS-CoV-2 infections among business associates in Germany was traced to a single pre-symptomatic individual [18]. In a more large-scale scenario, identification and rapid isolation of asymptomatic SARS-CoV-2 positive individuals led to quick decline in the number of new cases in an Italian village [13], suggesting that asymptomatic individuals were responsible for at least some of the spread of the virus. This is supported by a modeling study of transmission in China that suggests that ∼86% of infections were caused by asymptomatic or unreported cases [9]. These studies indicate that asymptomatic individuals could be a factor in the spread of SARS-CoV-2 and should be considered when predicting the scope of the epidemic and the effectiveness of mitigation strategies.

Mathematical modeling investigations of the role of asymptomatics during an epidemic have previously been done for other infectious diseases [19–26]. These models have estimated the proportion of asymptomatic individuals [22, 23, 25, 26] and have shown how changes in the proportion of asymptomatic individuals might change the course of the epidemic [19, 23, 24] or affect mitigation strategies such as quarantine [23] and vaccination [20, 21]. In the current SARS-CoV-2 pandemic, isolation and social distancing are the primary weapons in stopping the spread of the infection. In order to estimate how effective these strategies will be, we will need a better understanding of the role of asymptomatic individuals in SARS-CoV-2 spread and the effect the proportion and relative infectiousness of asymptomatics have on the time course of the epidemic.

In this paper, we study a compartmental epidemic model that includes asymptomatic infections to determine the role that asymptomatic individuals might play in the spread of SARS-CoV-2. We find that the relative infectiousness of asymptomatic individuals has more of an impact on the time course and size of the epidemic than the proportion of asymptomatic individuals, but that the proportion of asymptomatic individuals has a bigger impact on mortality. We apply our model to data from SARS-CoV-2 epidemics in California, Florida, New York, and Texas, finding that a large number of infections in these states are unreported and that relaxing social distancing measures too early will cause a rapid spike in infections driven in part by these hidden infections.

## Materials and methods

### Mathematical model

We use a compartmental model that includes asymptomatic infections with assumptions geared towards modeling the current SARS-CoV-2 pandemic,

$$\begin{array}{c} \begin{array}{cc} \frac{dS}{dt} & =-\frac{\beta }{N}SI-r\frac{\beta }{N}SA\\ \frac{dA}{dt} & =p\frac{\beta }{N}(SI+rSA)-kA\\ \frac{dI}{dt} & =\left(1-p\right)\frac{\beta }{N}(SI+rSA)-\left(\alpha +\delta \right)I\\ \frac{dR}{dt} & =kA+\alpha I\\ \frac{dD}{dt} & =\delta I.\; \end{array} \end{array}$$ (1)

where *S* is the pool of susceptible individuals, *A* are individuals infected asymptomatically, *I* are individuals infected symptomatically, *R* are recovered individuals, and *D* are those who have died. We assume that the course of the epidemic is short compared to the human lifespan and do not include births or deaths from other causes, instead assuming that the total population, *N* = *S* + *A* + *I* + *R* + *D* stays constant over the course of the epidemic. Upon exposure to the virus, some fraction (given by *p*) of people become asymptomatically infected; the remaining people become symptomatically infected. We assume that the asymptomatics can infect susceptible individuals, but with a different infection rate, determined by the proportionality constant *r*. We assume that asymptomatic individuals will all recover (after an average time 1/*k*), but that symptomatically infected individuals are removed either through recovery or hospitalization/isolation (at rate *α*) or die (at rate *δ*).

### Data fitting

To apply our model to the current pandemic, we use four US states as examples: California, Florida, New York, and Texas. We use data from the state health departments of California, Florida, New York, and Texas that tracks the cumulative number of infected individuals and the cumulative number who have died through April 17, 2020. Data is included in S1 File. We assume that the epidemic starts with a single symptomatic individual, *I*(0) = 1 and that there are no asymptomatic, recovered or dead (*A*(0) = *R*(0) = *D*(0) = 0). Since we do not know the time at which the infected individual arrived to start each epidemic, we introduce a free parameter, *T*_*d*_, to shift the data in time and set the appropriate start time of the infection. We fix the death rate of symptomatic individuals to *δ* = 0.056/d based on mean time from symptom onset to death [27]. Since our data includes the cumulative number of infected individuals, we define a variable *C*, where $\frac{dC}{dt}=\left(1-p\right)\frac{\beta }{N}(SI+rSA)$ tracks the cumulative number of symptomatically infected individuals and fit that to the cumulative infected individuals. Early in the pandemic, testing was primarily limited to individuals who displayed symptoms, so the case count in this data set should primarily consist of symptomatic individuals. We simultaneously fit both the infected and dead to the model by minimizing the weighted sum of square residuals (SSR) using the Nelder-Mead algorithm implemented in Octave. Confidence intervals are determined through bootstrapping with 1000 replicates.

## Results

### Effect of asymptomatics on infection size and deaths

We explored the effect of asymptomatics on the course of the infection using a base set of parameters given in Table 1. We used the population of the United States for the population size and used estimates for the remaining parameters from the literature. Infections are initiated with a single symptomatic infected individual. The infection rate was taken from a model fit to data of coronavirus infections from China [9]; they estimate infection rates before and after travel bans and social distancing measures were put in place. We use both values in our simulations. The mean time from symptom onset to death is 18 d [27] or *δ* = 0.056/d. We use a removal rate of *α* = *k* = 0.143/d based on data suggesting the mean time from symptom onset to hospitalization is 7 d [27]. Although many people who are hospitalized eventually recover, once they are hospitalized they are not contributing much to widespread community transmission.

**Table 1:** Model parameters.

Parameter	Value
*N*	320,000,000
*β*	1.12/d, 0.52/d ^a^
*δ*	0.056/d ^b^
*k*	0.143/d ^b^
*α*	0.143/d ^b^

^*a*^ Taken from [9].

^*b*^ Taken from [27].

**Fig 1:**
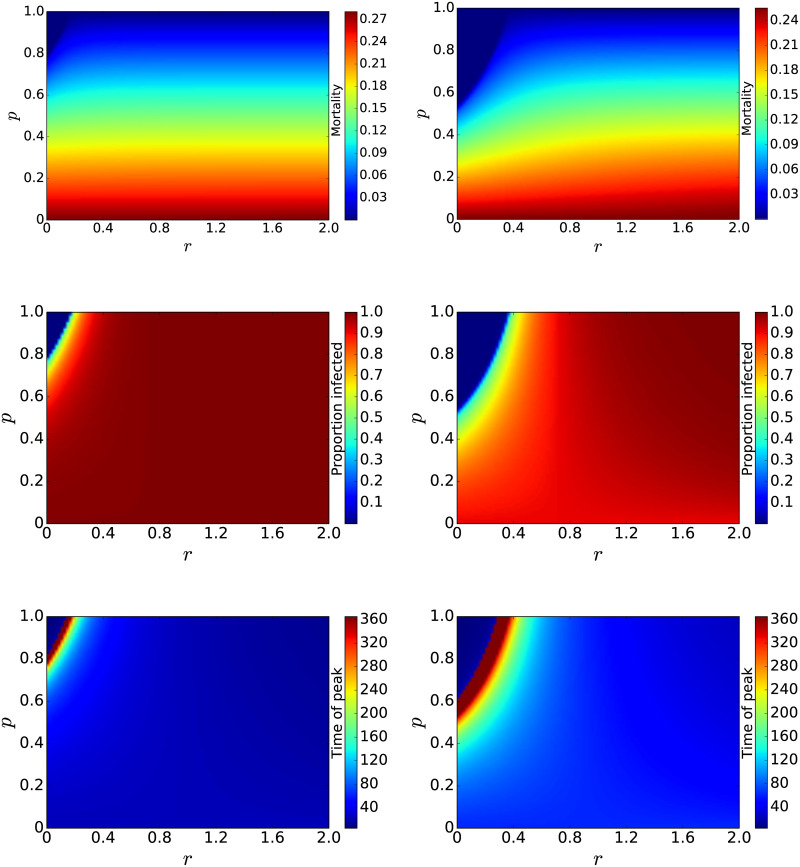
Effect of asymptomatic individuals on case-fatality ratio (top row), size of the epidemic (center row), and time of peak of the epidemic (bottom row). The left column shows results for a high infection rate and the right column shows results for a low infection rate. The relative infectivity of asymptomatics (*r*) has little effect on mortality, but the mortality decreases as the proportion of asymptomatics increases. Both the time of peak and the size of the epidemic remain fairly constant until the threshold for the epidemic is reached.

To study the role of asymptomatic individuals in an epidemic, we varied both *r* (from 0 to 2) and *p* (from 0 to 1) and measured the case-fatality ratio, the size of the epidemic, and the time of peak of the epidemic. The case-fatality ratio is defined as the number of dead divided by the total number infected. The size of the epidemic is measured here as the proportion of the total population who become infected, either symptomatically or asymptomatically. Results are shown as contour plots in Fig 1 for both high (left column) and low (right column) infection rates. We see that the relative infectivity (*r*) of asymptomatic individuals has little effect on the case-fatality ratio, remaining essentially constant for all values of *r* except when *p* is near one. In the upper left corner, there is a pocket where the mortality is zero; this is particularly evident for the lower infection rate. This pocket is more clearly defined for the epidemic size and the time of peak. Both the epidemic size and the time of peak are fairly constant until we approach the upper left hand corner where there is a rapid decrease in the epidemic size and a rapid increase in the time of peak. This boundary defines a threshold for the epidemic—above this boundary, the epidemic simply dies off. This boundary is determined by the basic reproduction number *R*_0_ for the epidemic. *R*_0_ for this model is given by

$$R_{0}=\frac{pr\beta }{k}+\frac{(1-p)\beta }{\alpha +\delta }.$$ (2)

*R*_0_ here has two components: the first term, $R_{a}=\frac{pr\beta }{k}$, represents spread due to asymptomatic individuals, while the second term, $R_{s}=\frac{(1-p)\beta }{\alpha +\delta }$ represents spread due to symptomatic individuals.

**Fig 2:**
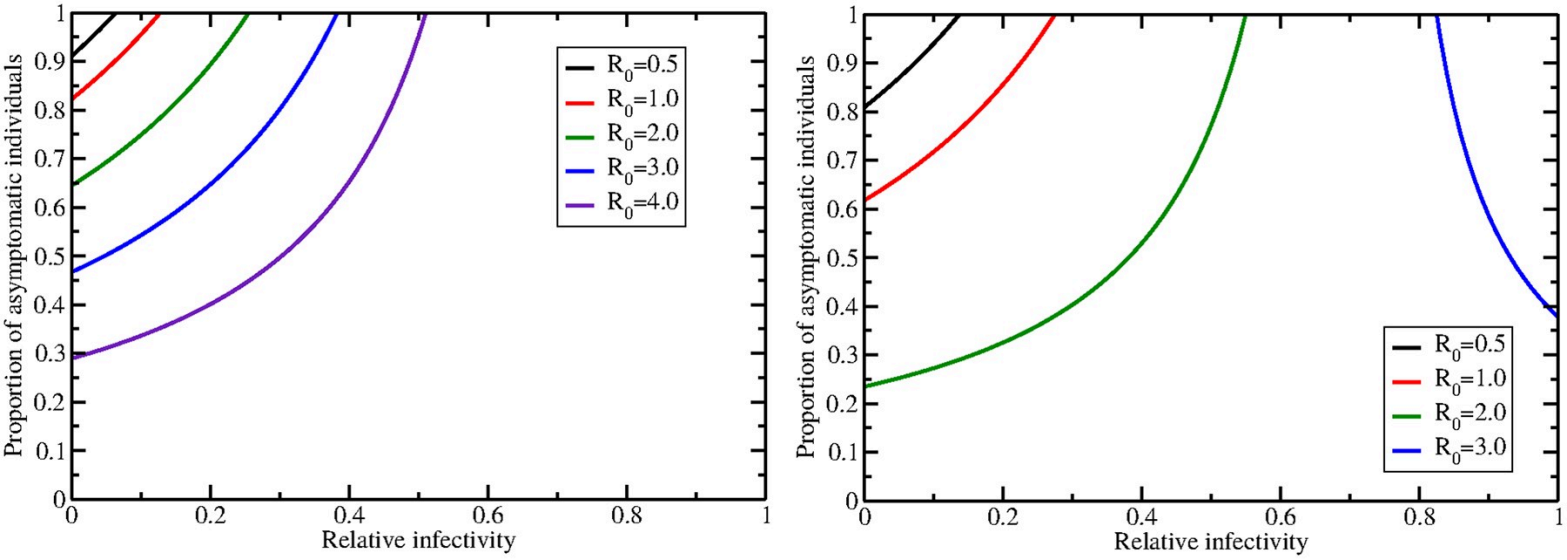
Trade-off between *p* and *r* to achieve a particular basic reproduction number for a high infection rate (left) and low infection rate (right).

Estimates of the basic reproduction number for SARS-CoV-2 outbreaks in various locations range from 0.5–4 [28], however, Fig 1 includes combinations of parameters leading to *R*_0_ values much larger than 4, so are likely biologically unrealistic. It might be more interesting to understand the trade-off between the proportion of asymptomatic infections and the relative infectivity in contributing to a particular value of *R*_0_. We fixed *R*_0_ to values within the known range for SARS-CoV-2 and plotted the values of *p* and *r* that will produce particular values of *R*_0_ for both the high (Fig 2 (left)) and low (Fig 2 (right)) values of infection rate. Solving for *p* in the *R*_0_ equation gives the relationship between *p* and *r*,

$$p=\frac{R_{0}k\left(\alpha +\delta \right)-k\beta }{r\beta (\alpha +\delta )-k\beta }.$$ (3)

When the infection rate is high, a high proportion of asymptomatics and low relative infectivity is required for low values of *R*_0_. The range of possible *p* and *r* values expands as *R*_0_ increases, with the relationship indicating that higher values of *p* require higher values of *r* for a particular *R*_0_. When the infection rate is low, we see a slightly different relationship between *p* and *r* for high *R*_0_—when *R*_0_ = 3, increasing *r* values require lower values of *p* for a particular *R*_0_. This change in behavior occurs because we have crossed an asymptote at *r* = *k*/(*α* + *δ*) (∼0.72 for these parameters). Note that the *R*_0_ = 4 curve does not appear in this plot because there are no biologically reasonable combinations of *p* and *r* that will result in *R*_0_ values of 4.

**Fig 3:**
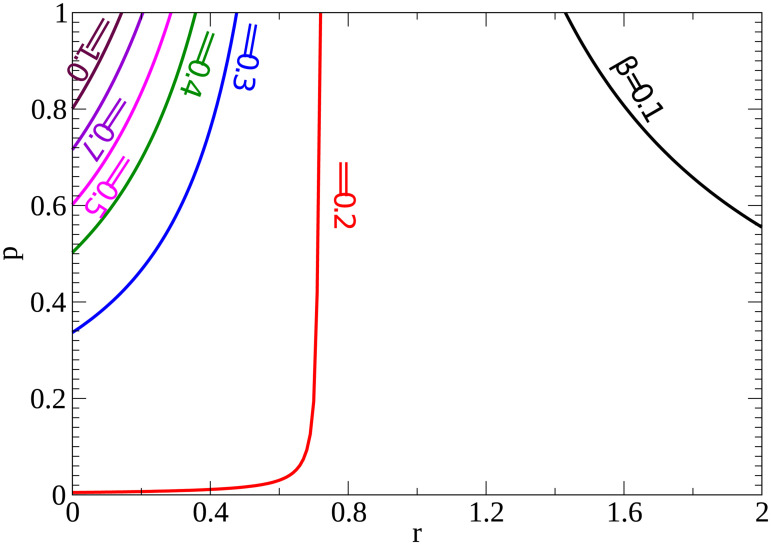
Effect of infection rate on epidemic threshold. The contours indicate the points where *R*_0_ = 1; to the left of each curve *R*_0_ < 1 and there will be no epidemic.

In the current COVID pandemic, public health authorities have been attempting to lower the infection rate through social distancing measures, so we more closely examined the dependence of the epidemic threshold on infection rate. Fig 3 shows the *R*_0_ = 1 (the epidemic threshold value) curves for different values of infection rate (*β*). *R*_0_ < 1 is to the left of each curve. We see that as the infection rate is lowered, there are more combinations of *r* and *p* that result in no epidemic. That is, the general infection rate is just not high enough to support spread of the infection no matter what the proportion of asymptomatic individuals or their relative infectivity.

### Application to SARS-CoV-2

**Fig 4:**
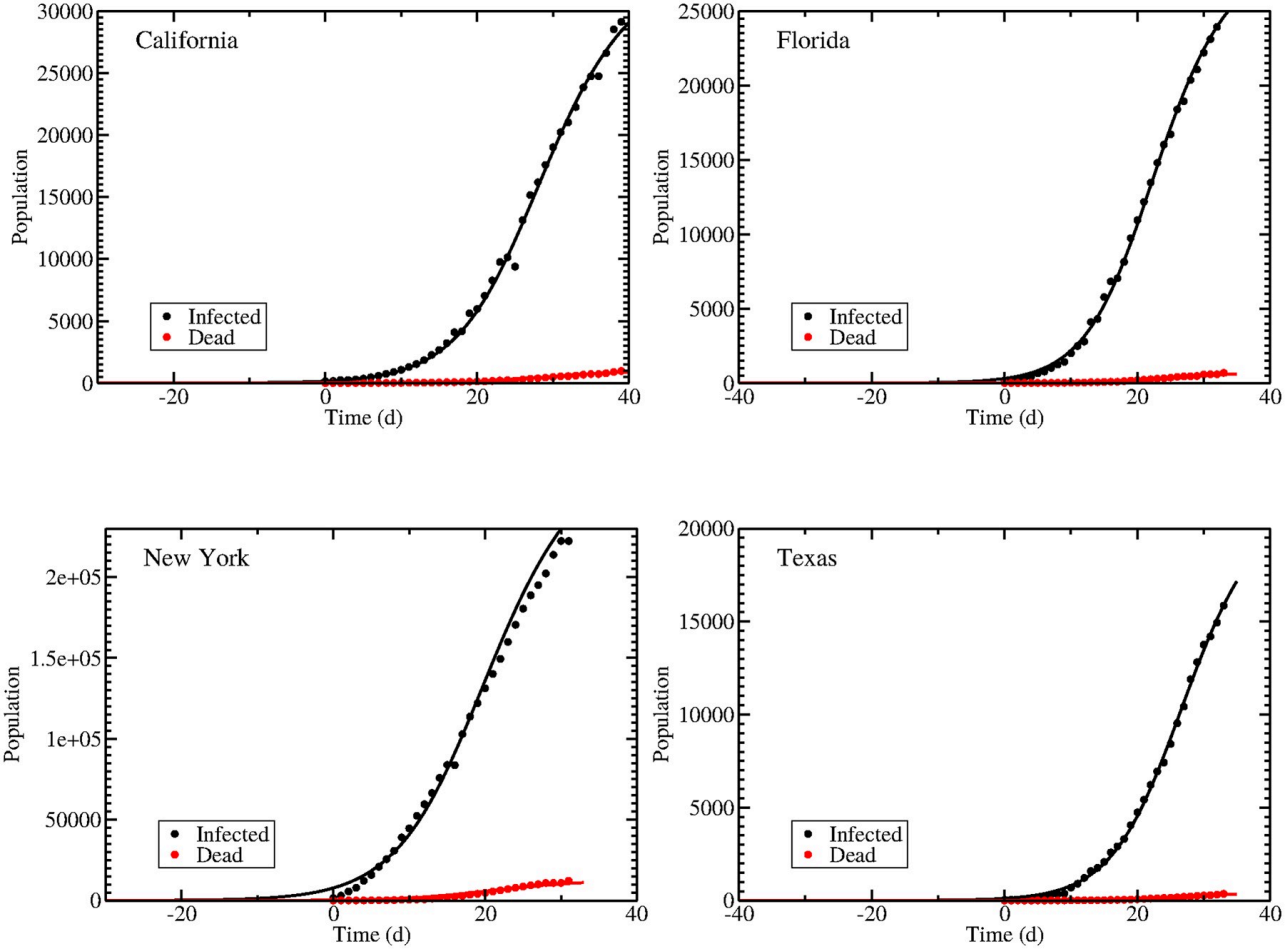
Data and model fits for SARS-CoV-2 epidemics in California (upper left), Florida (upper right), New York (bottom left), and Texas (bottom right). Best fit parameters are given in Table 2.

We fit the model to data from four states: California, Florida, New York, and Texas. Data and model fits to the data are shown in Fig 4. Best fit parameter values with 95% confidence intervals are given in Table 2. Parameter correlation and parameter distribution plots are included in S1 File. All four of these states have implemented some form of social distancing for a period of time, and the majority of the data is taken after these measures are in place. All four states have *R*_0_ values near 2. Estimates of the *R*_0_ for SARS-CoV-2 before changes in social behavior in China run between 2 and 4.25 [29–31], typically falling below 1 when complete lockdowns are imposed [28]. The *R*_0_ values found here are on the low end for unmitigated SARS-CoV-2, suggesting that the social distancing measures are having some effect, but the measures taken in these states have not driven *R*_0_ below the threshold value of 1. Interestingly, the estimated *R*_0_ values are almost entirely determined by the basic reproduction number for asymptomatic spread in all four states, indicating that the spread of the infection is largely driven by asymptomatic individuals, at least in the early stages of the pandemic. The large *R*_*a*_ value is almost entirely determined by the high proportion of asymptomatic individuals—about 99% for all four states. This is somewhat higher than the 86% unreported cases estimated in China [9] and might be indicative of the limited testing availability in the United States early in the pandemic. The undercounting of symptomatic infections is particularly problematic since each symptomatic person incorrectly classified as asymptomatic because they weren’t tested might appear as several asymptomatic people in order to account for the difference in infectivity between the two groups. The high proportion could also be in part due to the lack of a pre-symptomatic phase in this model; recent studies have shown that symptomatic people can infect others during the pre-symptomatic phase [32–34] and within this model would be considered part of the asymptomatic population. The relative infectivity of asymptomatic patients ranges from ∼6–15%, so the high basic reproduction number for this group is driven by the large number of patients rather than their ability to easily infect others. The infectivity values for all four states are high, so based on the analysis of Fig 2, we expect that there are few combinations of *p* and *r* that will result in *R*_0_ values near 2 and that this can only happen if *p* is large and *r* is small.

**Table 2:** Best fit parameters and 95% confidence intervals for COVID epidemics in California, Florida, New York, Texas.

Parameter	California	Florida	New York	Texas
*β* (/d)	3.48 (3.46–3.49)	4.97 (4.85–5.08)	2.53 (2.40–2.67)	5.51 (5.40–5.61)
*r*	0.112 (0.110–0.114)	0.0734 (0.0702–0.0772)	0.153 (0.137–0.170)	0.0635 (0.0617–0.0658)
*p*	0.999 (0.999–0.999)	0.998 (0.998–0.998)	0.980 (0.979–0.981)	0.999 (0.999–0.999)
*k* (/d)	0.198 (0.197–0.199)	0.161 (0.154–0.174)	0.212 (0.196–0.229)	0.154 (0.149–0.161)
*α* (/d)	1.76 (1.40–2.33)	2.33 (1.76–2.78)	1.17 (0.968–1.34)	2.69 (1.97–3.25)
*T*_*d*_ (/d)	55.2 (52.8–56.0)	53.7 (52.0–56.1)	63.6 (60.5–67.3)	53.8 (51.6–55.6)
SSR	37.7 (14.0–98.3)	19.1 (8.51–46.0)	378 (88.5–446)	8.58 (7.71–24.7)
*R*_0_	1.97 (1.94–2.00)	2.26 (2.14–2.33)	1.83 (1.79–1.86)	2.28 (2.20–2.35)
*R*_*a*_	1.96 (1.94–2.00)	2.25 (2.13–2.33)	1.79 (1.74–1.82)	2.27 (2.20–2.35)
*R*_*s*_	0.00213 (0.00161–0.00270)	0.00336 (0.00286–0.00443)	0.0414 (0.0367–0.0494)	0.00182 (0.00152–0.00254)

**Fig 5:**
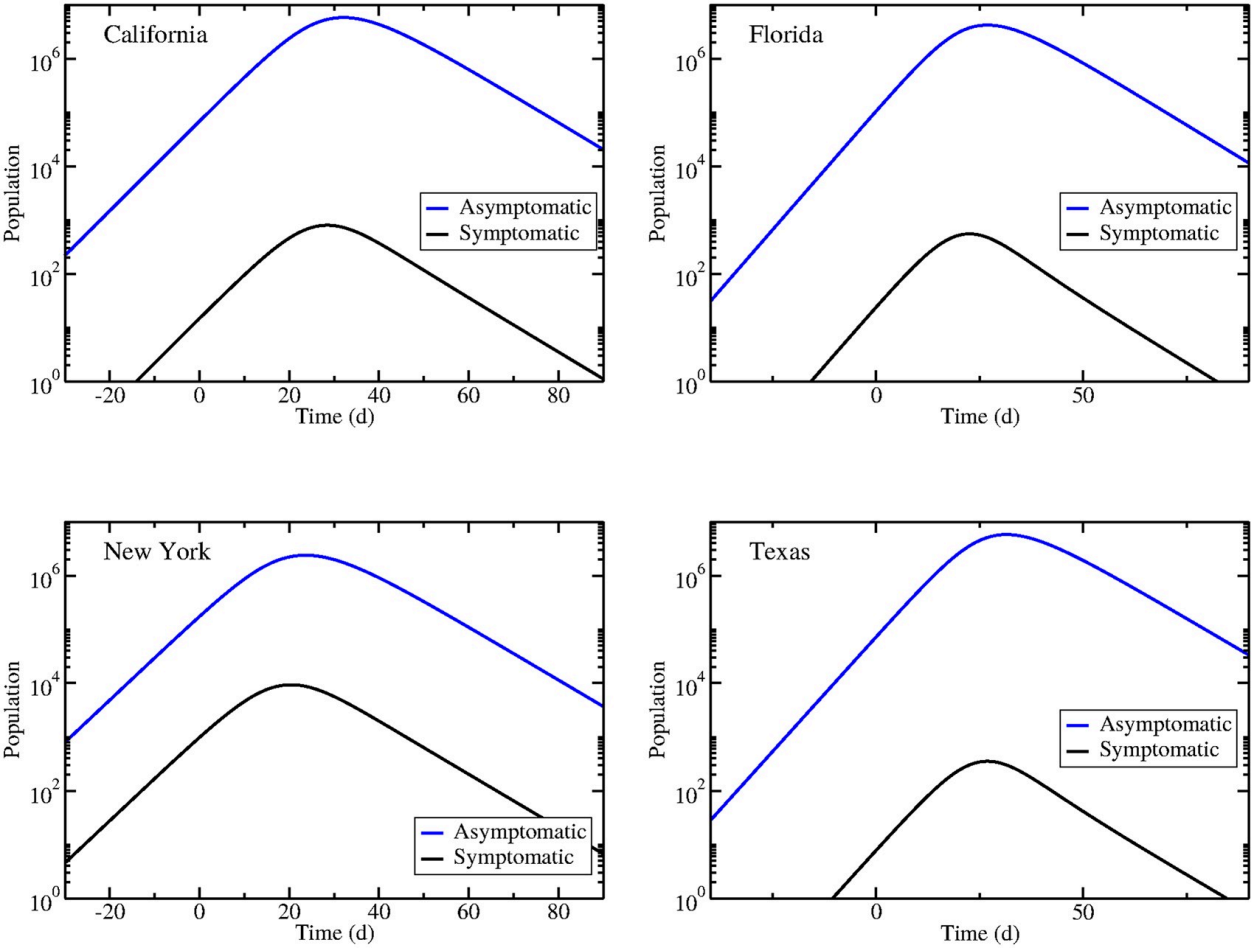
Estimated asymptomatic dynamics during epidemics in California (upper left), Florida (upper right), New York (bottom left), and Texas (bottom right).

Fig 5 shows the time course of asymptomatic individuals and symptomatic individuals for epidemics in all four states. In all cases, at the peak of the epidemic, there are far more asymptomatic individuals than symptomatic individuals. This is due not only to the high proportion of infections that are asymptomatic, but is also caused by a faster removal of symptomatic individuals as compared to asymptomatic individuals. While it might seem unusual that asymptomatic individuals are recovering slower than symptomatic individuals, given the heightened awareness of transmission of infectious diseases as well as the stay-at-home orders in these states, symptomatic individuals are likely to isolate themselves upon symptom onset, effectively removing themselves from participating in further transmission. While not true recovery, from the standpoint of the model, these symptomatic people are classified as recovered. Therefore, asymptomatic individuals participate in transmitting the infection for a longer period of time and in greater numbers than asymptomatic individuals. It is also notable that the peak in asymptomatic infections occurs slightly after the peak in symptomatic infections. This could have repercussions for decisions on when to relax social distancing measures since we will observe a decline in infections while asymptomatic infections are still increasing.

We also estimated the time at which the initial infected individual initiated the infection in each state. Using our estimates of *T*_*d*_, we can calculate the estimated date of the start of the epidemic. For all four states, we find that the epidemics were initiated sometime in January 2020. For California, this is as early as January 15, while for Florida the estimated infection start date is January 14. New York has the earliest estimated start date of January 11, while the latest is in Texas on January 24.

### Changing the infection rate

**Fig 6:**
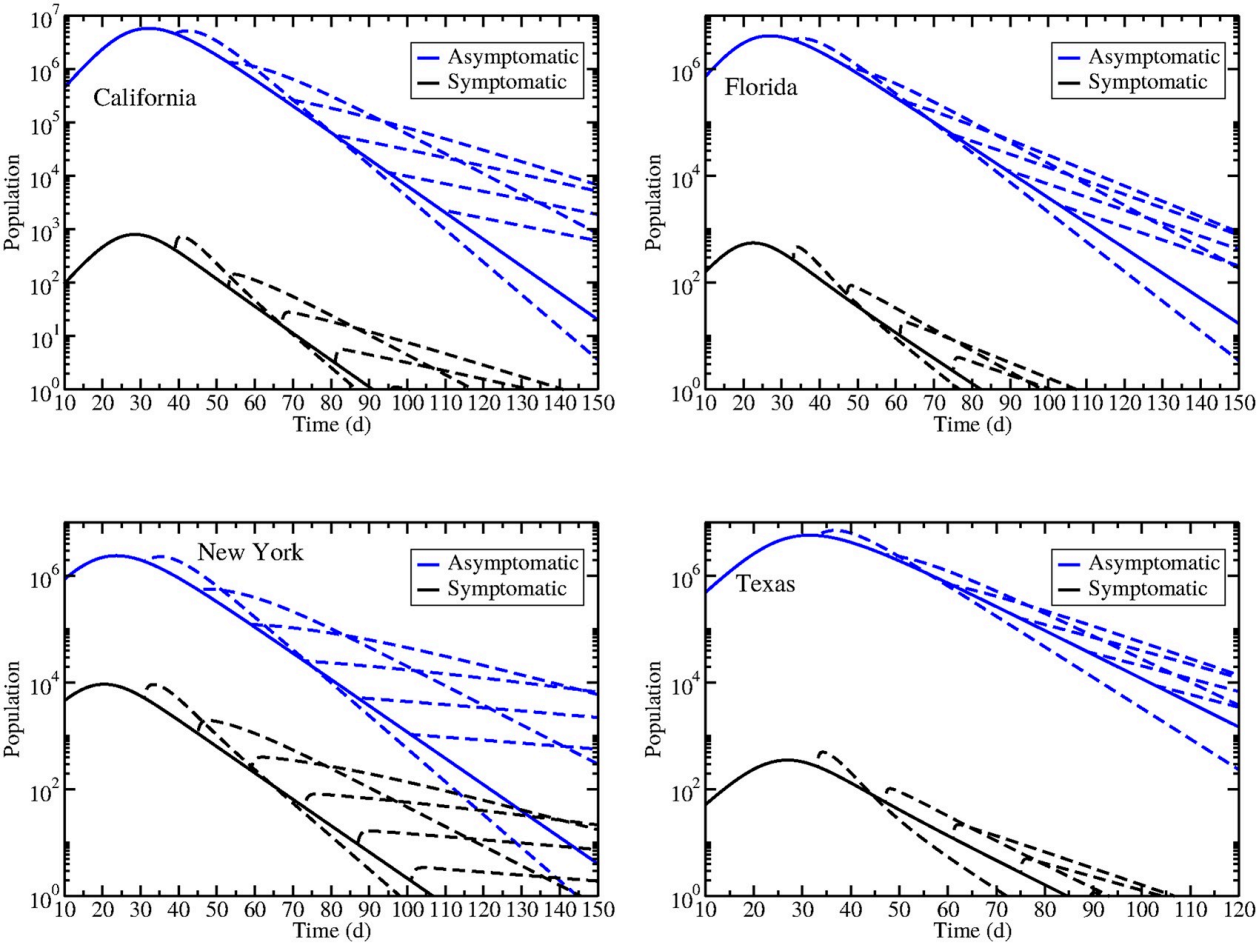
Estimated dynamics during epidemics when social distancing measures are relaxed. Shown are epidemics in California (upper left), Florida (upper right), New York (bottom left), and Texas (bottom right) where each dashed line shows the trajectory if social distancing measures are relaxed at different two week intervals.

Strict stay-at-home measures have severe economic implications and are difficult to maintain [35, 36], so government officials want to relax social distancing measures as early as possible. Given the large number of asymptomatic individuals predicted by our model, as well as the fact that the asymptomatic peak is slightly later than the symptomatic peak, we decided to investigate model predictions of what happens as social distancing measures are relaxed. Based on a previous modeling study, we assume that stay-at-home orders cut the infection rate *β* in half [9], so double the infection rate at two week intervals starting on the last day of our data (April 17, 2020). Results are shown in Fig 6 with the solid line indicating the original epidemic where social distancing was not relaxed, and dashed lines indicating predicted epidemics when social distancing is relaxed. We see that there is an immediate spike in both symptomatic and asymptomatic infections upon reopening and that the spike becomes smaller as reopening is delayed. Interestingly, there is also a change in the decay rate of the epidemic as the re-opening date is changed. Earlier re-openings have a larger spike in cases, but also exhibit a more rapid decline in cases. In fact, if all four states re-opened immediately, the decay rate would be faster than if the states continued with social distancing measures indefinitely.

**Fig 7:**
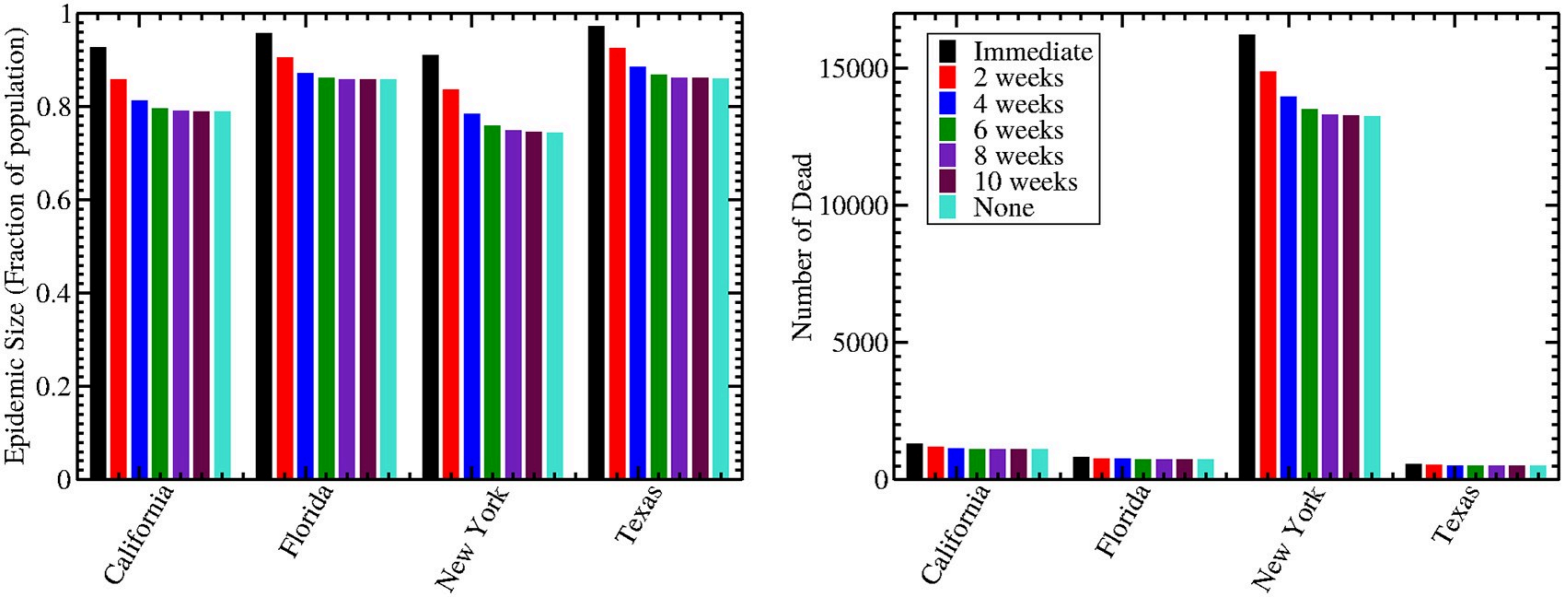
Estimated epidemic size (left) and number of dead (right) for different reopening timelines. We examined the effect of relaxing social distancing measures at different times from the end of our data (April 17, 2020).

To further quantify the effect of reopening at different times, we found the final death toll and the total size of the epidemic for each reopening scenario. Results are shown in Fig 7. In all cases, delaying re-opening lowers the size of the epidemic as well as the number of fatalities. Even though immediate re-opening might result in a faster resolution to the epidemic, this comes at the cost of more illness and deaths. For California, Florida, and Texas, both the total epidemic size and the number of fatalities increase with any re-opening within the next 10 weeks, as compared to no relaxation of social distancing measures for the duration of the epidemic.

## Discussion

We analyzed a compartmental model that includes asymptomatic infections finding that the relative infectiousness of asymptomatic individuals plays a larger role in changing the time course and size of the epidemic while the proportion of asymptomatic infections plays a larger role in determining mortality. Yet testing in many countries is limited to people who are symptomatic, meaning that there is no data on the number of asymptomatic infections, so mathematical models cannot properly incorporate asymptomatic individuals. This not only hampers the ability of the model to accurately predict the time course of the epidemic, but could also lead to inaccurate predictions of the effect of isolation [23] and contact-tracing mitigation strategies. If a vaccine is eventually developed, previous studies have shown that the number of asymptomatic infections also alters the effectiveness of vaccination strategies [20, 21].

When we fit our model to data from the SARS-CoV epidemic in four states, we found that there were far more asymptomatic individuals than symptomatic individuals at the peak of the epidemic in all four states. This is consistent with recent studies of antibody seroprevalence that suggest a 50-85 fold undercount of COVID-19 in Santa Clara County, California [37] and in Los Angeles [38]. Our model has two different mechanisms by which the number of asymptomatic individuals can rise to high numbers. If the proportion of infections that become asymptomatic is high (*p* close to 1), then as the number of infections rise, more of these will be asymptomatic. It is also possible to accumulate large numbers of asymptomatic individuals if symptomatic infections are removed from the epidemic, either through death, recovery, or isolation, faster than asymptomatic infections. This might be the case if symptomatic individuals are quickly tested and then isolated thereby being removed from further participation in the infection as might be the case for these four states since testing for SARS-CoV-2 in all these states at the time this data was taken, was limited to symptomatic individuals.

This also points to a limitation of this study. Testing for SARS-CoV-2 started slowly in the United States [39] and remains inadequate to this day, so there is no tracking of the number of asymptomatic cases and likely an undercount of symptomatic cases. Thus the asymptomatic compartment in this application of the model includes symptomatic patients who were unable to get tested. Our model also does not include a latent or pre-symptomatic phase, which also lumps people who likely have different infection rates into one compartment. This mixing of individuals who should be in separate compartments as well as the inadequate counting of symptomatic infections leads to error in our parameter estimates and an inability to uniquely identify all parameters of the model [40]. Correlation plots included in S1 File show that there are correlations between some parameter estimates, although there was no consistent pattern on which parameters were correlated between the four data sets used. Adequate testing protocols that capture both symptomatic and asymptomatic infections will allow better parameterization of the model. Additional data, such as tracking the number of recovered individuals would also help constrain parameters. While some state public health agencies are reporting the number of recovered, this number is really only reflective of symptomatic patients who have been hospitalized and released. Symptomatic patients who remain at home are not being tracked, so their recovery is not recorded. Accurately tracking the course of all aspects of the epidemic, particularly in its early phase [41], is crucial for proper parameterization of mathematical models and will lead to more accurate model predictions.

Despite these limitations, fitting of our model to the data has resulted in some interesting parameter estimates. The basic reproduction number appears to be robust to limitations of parameter identifiability [42]. We found a basic reproduction numbers around 2 for all four states. This is at the lower end of SARS-CoV *R*_0_ estimates in the absence of any behavioral changes [29–31]. These low values of *R*_0_ likely reflect the effect of social distancing measures in the US, although these measures have not driven *R*_0_ below 1 as a full lockdown would [28]. However, a recent study [43] indicates that SARS-CoV-2 oubreaks with *R*_0_ < 1.5 can be contained with 50% contact tracing. Even for *R*_0_ up to 2.5, contact tracing only needs to capture 70% of infections to be effective.

## Conclusion

While the epidemiological model used here is not as complex as some others that have been suggested for SARS-CoV-2, it still provides insight into a key aspect of SARS-CoV-2 transmission. Our model shows that asymptomatic infections can change the size and lethality of an epidemic even if they are not necessarily a large proportion of the infections. For the SARS-CoV epidemics examined here, the model predicts that there are far more asymptomatic or unreported cases at the peak of the infection, suggesting that there might be widespread community transmission if stay-at-home orders are relaxed too early.

## Supporting information

S1 File File containing epidemiological data and parameter correlation and distribution plots. (PDF)
