# Supplementary material for: Modeling the role of asymptomatics in infection spread with application to SARS-CoV-2
Source: PLoS One. 2020 Aug 10;15(8):e0236976. doi: 10.1371/journal.pone.0236976 (PMC7416915; doi:10.1371/journal.pone.0236976)
Supplement: S1 File — (PDF) [file pone.0236976.s001.pdf]

Supplement for "Modeling the role of asymptomatics in infection spread with application to SARS-CoV2"

Hana M. Dobrovolny

In this supplement we present graphs showing the parameter distributions determined from bootstrapping and include the data used to estimate parameters for the SARS-CoV-2 epidemic in California, Florida, New York, and Texas.

Table 1: Epidemiological data for California.

Day	Cumulative Infected	Cumulative Dead
0	148	2
1	161	3
2	246	4
3	249	4
4	288	4
5	355	4
6	465	6
7	578	11
8	734	14
9	888	17
10	1063	19
11	1292	24
12	1532	29
13	1838	33
14	2272	40
15	2672	52
16	3222	67
17	4092	70
18	4179	83
19	5624	115
20	5970	124
21	7037	142
22	8275	168
23	9757	206
24	10143	236
25	9394	203
26	13139	289
27	15156	344
28	16195	372
29	17595	430
30	19020	485
31	20229	538
32	21023	556
33	22257	602
34	23851	674
35	24730	721
36	24745	721
37	26598	790
38	28523	890
39	29129	973

Table 2: Epidemiological data for Florida.

Day	Cumulative Infected	Cumulative Dead
0	139	3
1	172	5
2	222	6
3	335	7
4	440	8
5	662	10
6	772	10
7	1017	12
8	1243	16
9	1430	18
10	1999	23
11	2485	29
12	2791	34
13	4094	56
14	4302	56
15	5775	71
16	6826	85
17	7041	87
18	8138	128
19	9748	163
20	10951	191
21	12182	221
22	13468	254
23	14800	296
24	16021	323
25	16718	354
26	18387	419
27	18932	438
28	20356	461
29	21071	470
30	22199	571
31	23110	591
32	23931	591
33	25439	686

Table 3: Epidemiological data for New York.

Day	Cumulative Infected	Cumulative Dead
0	1560	16
1	3081	29
2	5673	36
3	7883	35
4	12264	56
5	15949	117
6	21067	156
7	25811	233
8	31140	327
9	39531	485
10	45120	485
11	53082	728
12	60251	738
13	67715	1218
14	77345	1550
15	85817	1940
16	86085	2373
17	105798	2935
18	117271	3565
19	126190	4159
20	135997	4758
21	145636	5558
22	155669	6268
23	167004	7067
24	178356	7844
25	189085	8627
26	198079	9385
27	205087	10056
28	213042	10834
29	224613	10834
30	233118	10834
31	234476	12192

Table 4: Epidemiological data for Texas.

Day	Cumulative Infected	Cumulative Dead
0	38	0
1	51	0
2	59	0
3	61	1
4	77	1
5	109	2
6	234	4
7	309	5
8	343	5
9	370	5
10	698	9
11	902	11
12	1214	14
13	1582	18
14	1754	23
15	2079	27
16	2586	34
17	2915	38
18	3307	41
19	4055	58
20	4739	70
21	5420	90
22	6215	105
23	6939	127
24	7416	140
25	8416	154
26	9530	177
27	10429	199
28	11897	226
29	12815	254
30	13755	271
31	14193	287
32	14942	318
33	15856	364

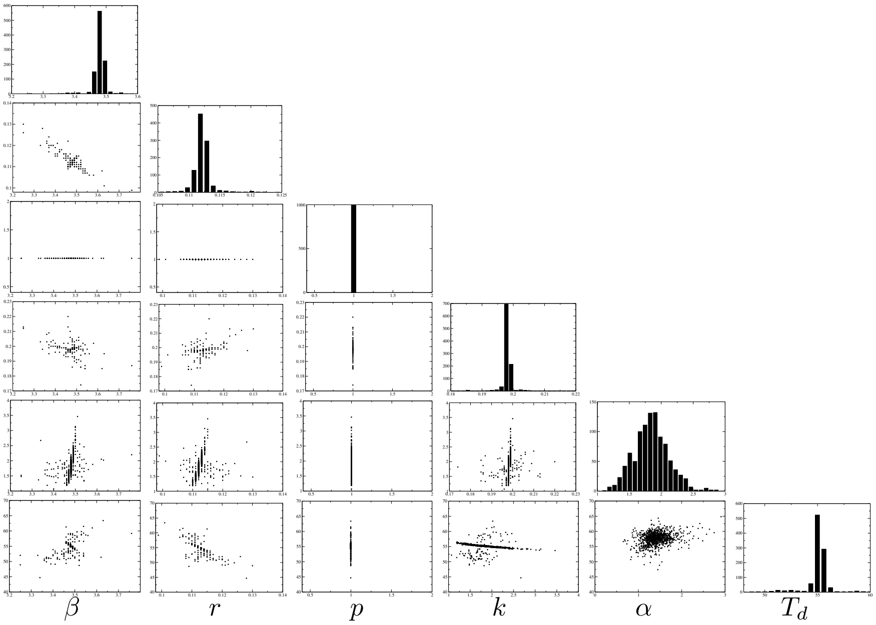

Figure 1: Parameter correlation plots and parameter distributions for fits to the epidemiological data from California.

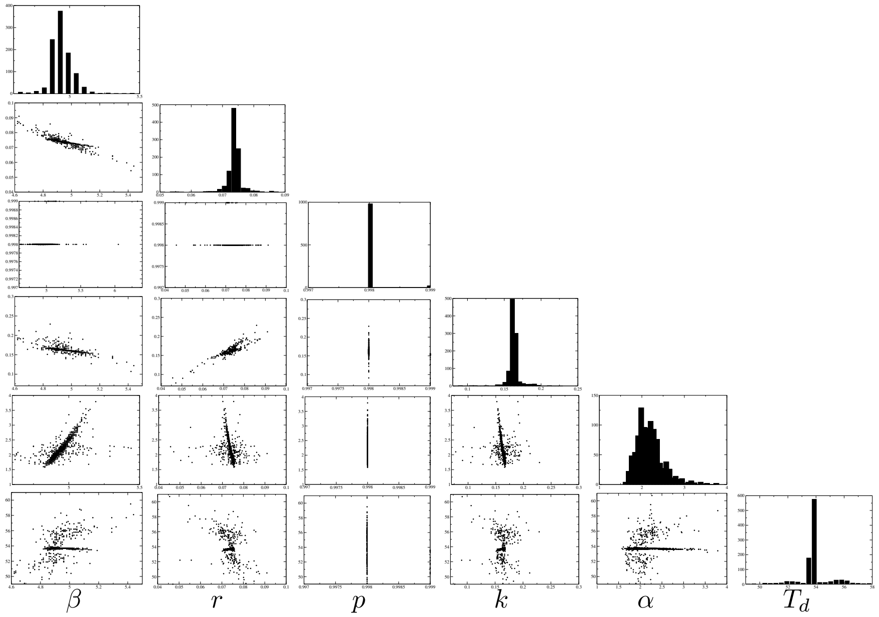

Figure 2: Parameter correlation plots and parameter distributions for fits to the epidemiological data from Florida.

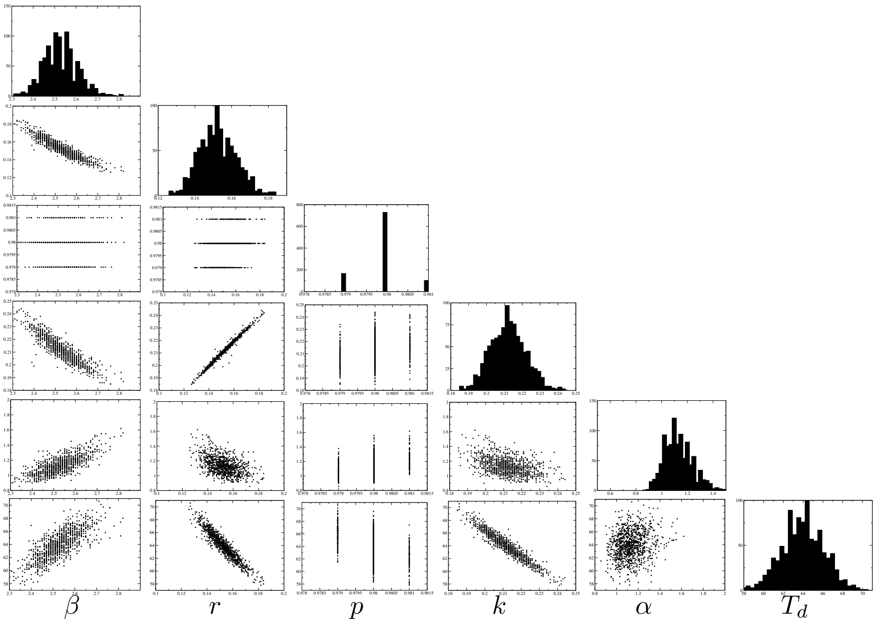

Figure 3: Parameter correlation plots and parameter distributions for fits to the epidemiological data from New York.

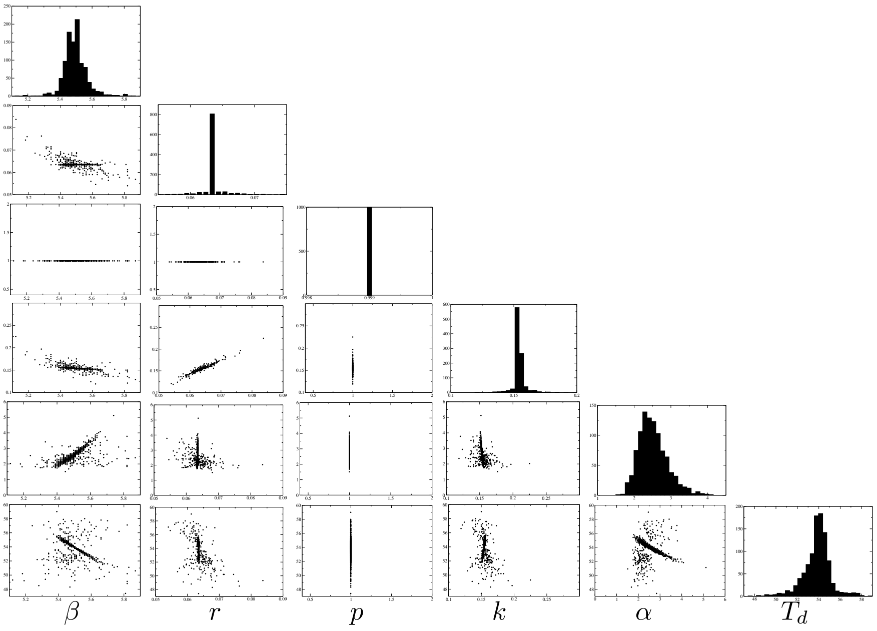

Figure 4: Parameter correlation plots and parameter distributions for fits to the epidemiological data from Texas.
